# Monomeric and Dimeric Carboxylic Acid in Crystalline Cavities and Channels of Delta and Epsilon Forms of Syndiotactic Polystyrene

**DOI:** 10.3390/polym13193330

**Published:** 2021-09-29

**Authors:** Antonietta Cozzolino, Guglielmo Monaco, Christophe Daniel, Paola Rizzo, Gaetano Guerra

**Affiliations:** Dipartimento di Chimica e Biologia “A. Zambelli” and INSTM Research Unit, Università degli Studi di Salerno, Via Giovanni Paolo II, 13284084 Fisciano, Italy; gmonaco@unisa.it (G.M.); cdaniel@unisa.it (C.D.); gguerra@unisa.it (G.G.)

**Keywords:** nanoporous-crystalline forms, crystalline polymer hosts, low-molecular-mass guests, polarized FTIR, carbonyl stretching peaks, WAXD

## Abstract

Delta (δ) and epsilon (ε) co-crystalline forms of syndiotactic polystyrene with a carboxylic acid guest were obtained by sorption of liquid hexanoic acid in syndiotactic polystyrene films exhibiting delta and epsilon nanoporous-crystalline forms. The characterization study is facilitated by axially stretched syndiotactic polystyrene films, used both for polarized FTIR spectra and for WAXD fiber patterns. Particularly informative are two carbonyl-stretching FTIR peaks, attributed to monomeric and dimeric hexanoic acid. The dichroism of these carbonyl peaks indicates that both delta and epsilon phases are able to include hexanoic acid as isolated guest molecules, while only the epsilon phase is also able to include dimeric hexanoic acid molecules in its crystalline channels. The inclusion of both isolated and dimeric hexanoic acid species in the epsilon form crystalline channels produces extremely fast hexanoic acid uptakes by syndiotactic polystyrene epsilon form films.

## 1. Introduction

Co-crystalline (CC) forms between a polymeric host and low-molecular-mass guest molecules are known for many polymers [1]. For most host polymers, co-crystalline phases are formed only with a very limited number of guest molecules, which exhibit specific host–guest interactions. This occurs, for instance, for isotactic polystyrene [2,3], syndiotactic poly-p-methylstyrene [4,5], polyethyleneoxide [6,7], poly(vinylidene fluoride) [8,9], isotactic poly-4-methyl-1-pentene [10] and poly(L-lactic acid) (PLLA) [11,12,13]. As an example, the only guest molecules which have been described as suitable to form CC forms with PLLA are cyclopentanone, dimethylformamide, tetrahydrofuran and dioxolane [11,12,13].

The behavior of two polymers, syndiotactic polystyrene (s-PS) and poly(2,6-dimethyl-1,4-phenylene ether) (PPO), is completely different: they are able to form not only CC forms but also nanoporous-crystalline (NC) forms (δ [14,15,16,17,18] and ε [19] for s-PS and α and β for PPO [20,21]), i.e., crystalline forms whose density is lower than the corresponding amorphous phases. These kinetically stable NC forms are able to absorb large amounts of many different kinds of guest molecules, leading to corresponding CC forms. In fact, by using suitable procedures, stable CC forms of s-PS and PPO can be easily prepared with a large number of organic compounds, provided that their molecular volume is smaller than 0.2−0.3 nm^3^ [1,16]. Suitable guests are not only low-polarity aromatic and aliphatic compounds but also high-polarity organic compounds [22] like p-nitro-aniline, for instance [23].

This wide availability of guests has opened the possibility of applications of CC polymer films in many different fields, including fluorescence [24,25], photoreactivity [26], magnetism [27] and ferroelectricity [28]. CC s-PS films [29,30,31,32] and fibers [33] have also been proposed for antimicrobial applications, by using poorly polar antimicrobials as guests, such as carvacrol [29,32], eugenol, parabens [30] and hexanal [31].

In this paper, we study CC forms of s-PS with a carboxylic acid (hexanoic acid, HA) [34], which has an antimicrobial activity against *E. coli* and *S. aureus* [35], as well as an antifungal activity on *Botrytis cinerea* [36]. Our analysis is mainly based on axially stretched s-PS films, which are used both for Polarized FTIR spectra and for WAXD fiber patterns. Particularly informative are the carbonyl stretching FTIR peaks, whose position depends on the presence or absence of hydrogen bonds between acid guest molecules [34,37,38,39,40,41,42].

## 2. Experimental Section

### 2.1. Materials

Hexanoic acid (HA) is a carboxylic acid with T_m_ = −3 °C and T_b_ = 205 °C, with acid dissociation constant pKa = 4.8 at 25 °C. HA and all used solvents were supplied by Sigma-Aldrich (St. Louis, MO, USA) and used without any further purification.

Syndiotactic polystyrene is from Idemitsu (Xarec 90ZC) (Tokyo, Japan) and has a content of syndiotactic triads higher than 98%. The average molar weight is M_w_ = 140 kg·mol^−1^, with polydispersity M_w_/M_n_ = 2.0.

Amorphous s-PS films were obtained by a blown extrusion process using a melt temperature of 290 °C. The bubble was obtained using a blow-up ratio of 2.5 and a draw ratio of 8. Axial stretching of amorphous films was conducted with a dynamometer INSTRON 4301 (Norwood, MA, USA) at 105 °C, up to a draw ratio of 4.0. Axially oriented films with the NC δ form were obtained by immersion of axially stretched amorphous films in dichloromethane at room temperature for 1 night, followed by guest removal by acetonitrile sorption for 3 h. Axially oriented films with the dense γ form were obtained by annealing of δ form films at 170 °C for 1 h. Axially oriented films with the NC ε form were obtained by immersion of the axially oriented γ form film for 1 h in chloroform and then in acetonitrile at room temperature.

### 2.2. Techniques and Methods

Wide-Angle X-ray Diffraction (WAXD) patterns were collected by an automatic Bruker diffractometer (Billerica, MA, USA), with nickel filtered CuKα radiation.

Fourier transform infrared (FTIR) spectra were collected in the wavenumber range 4000–400 cm^−1^, and with a resolution of 2.0 cm^−1^, by a Vertex 70 Bruker spectrometer (Billerica, MA, USA), equipped with deuterated triglycine sulphate (DTGS) detector and a Ge/KBr beam splitter. The frequency scale was internally calibrated to 0.01 cm^−1^ using a He−Ne laser. To reduce the noise, 32 scans were signal averaged. Polarized infrared spectra were obtained by using a SPECAC 12000 wire grid polarizer.

Evaluation of the degree of crystallinity (*X_c_*) of the films was performed by an FTIR spectral subtraction procedure according to the following formula K = (l/l’)(1 − *X_c_*) [43], where K is the subtraction coefficient, l and l’ are the thickness of the sample and of an amorphous reference film. The ratio l/l’ was spectroscopically estimated from the absorbance ratio of a conformationally insensitive peak (at 1601 cm^−1^). The degree of crystallinity of all s-PS semicrystalline films of this paper is in the range 25–30%.

The guest content in the films was evaluated by the intensity of FTIR guest peaks, as calibrated by thermogravimetric (TGA) measurements. TGA scans were performed with a TG 209 F1 Netzsch (Oberfranken, Bavaria, Germany) at a rate of 10 °C/min, in the temperature range 25−350 °C.

The order parameter of the host crystalline phase (*S_h_*) was calculated by the formula:

(1) $$S_{h}=\frac{R-1}{R+2}$$

where *R* = A_//_/A_⊥_ is the dichroic ratio, and A_//_ and A_⊥_ are the measured absorbance for polarization plane parallel and perpendicular to the draw direction, respectively. This orientation factor is equal to zero for random crystallite orientation, while it is equal to +1 and −0.5 for orientation of all polymer chain axes of the crystallites, being parallel and perpendicular to the stretching direction, respectively. As all considered s-PS films exhibit crystalline phases (γ, δ and ε) with s(2/1)2 helical conformation, the host order parameter was evaluated by the dichroic ratio of the 572 cm^−1^ infrared peak. This “helical” peak is characterized by the transition moment vector parallel to the chain axes. The order parameter of the used, axially stretched γ, δ and ε films is 0.83, 0.88 and 0.84, respectively.

Polarized FTIR spectra of CC films also allow an analogous evaluation of the order parameter of HA guest molecules, with respect to the film stretching direction (*S_g_*):

(2) $$S_{g}=\frac{R-1}{R+2}$$

which was evaluated for all the dichroic HA peaks (located at 3443, 1751 and 1709 cm^−1^).

DFT calculations of frequency and intensity of infrared spectra of isolated monomer and dimer were performed with Gaussian 16 [44] at the APFD [45]/6−311+G(d,p) level.

## 3. Results and Discussion

### 3.1. δ and ε Co-Crystalline Forms of s-PS with Hexanoic Acid

In this section, we describe sorption of HA in s-PS films exhibiting the dense γ form or the NC δ and ε forms. The reported results mainly refer to axially stretched s-PS films, because they allowed us to obtain WAXD fiber patterns and Polarized FTIR spectra, which are the most suitable techniques to establish the possible formation of polymer CC phases [1].

**Figure 1:**
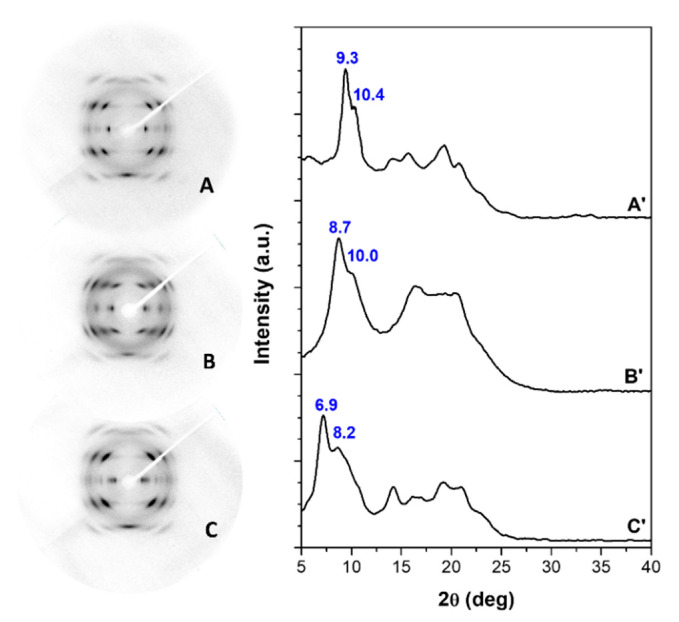
WAXD patterns of axially stretched s-PS films with a draw ratio of 4, exhibiting γ (**A**,**A’**), δ (**B**,**B’**) and ε (**C**,**C’**) crystalline phases: (**A**–**C**) 2D patterns; (**A’**–**C’**) equatorial profiles of the 2D patterns.

The WAXD patterns of s-PS films (axially stretched up to a draw ratio of 4 and with similar degrees of crystallinity, in the range 25–30%) that exhibit γ, δ and ε crystalline phases, are shown in Figure 1A–C, respectively.

Patterns A and A’ (mainly the equatorial peaks at 2θ = 9.3° and 10.4°) indicate the presence of the γ phase [46]. Patterns B and B’ (mainly the equatorial peak at 2θ = 8.7° and the shoulder at 2θ ≈ 10°) indicate the presence of the triclinic NC δ phase [17]. Patterns C and C’ (mainly the equatorial peaks at 2θ = 6.9° and at 2θ = 8.2°) indicate the presence of the orthorhombic NC ε phase [19].

HA uptake from these semicrystalline s-PS films was studied by FTIR spectra [34], as calibrated by TGA analyses.

**Figure 2:**
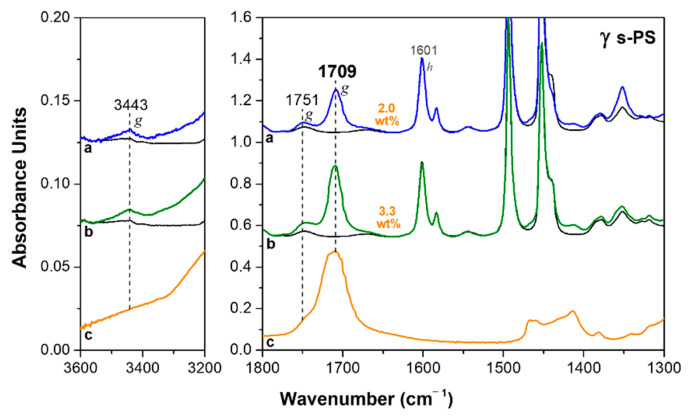
FTIR spectra of an axially oriented γ form s-PS film after immersion in: (**a**) HA at 70 °C for 12 h; (**b**) 40 wt% solution of HA in acetone at room temperature for 12 h, followed by acetone desorption at 50 °C for 1 h. The uptake of HA in the film (expressed as wt%) is indicated close to the spectra. The FTIR spectrum of liquid HA is shown for comparison in (**c**).

FTIR spectra of a 50 µm axially stretched γ form film, after sorption of HA in different conditions, are shown in Figure 2. The uptake of HA, as evaluated based on the intensity of the carbonyl stretching peak at 1709 cm^−1^ (Figure 2c), after 12 h of film immersion in liquid HA at 70 °C, is about 2 wt% (Figure 2a). The HA uptake can be increased by immersion of the film in solutions of the acid in volatile carrier solvents. For instance, after 12 h of film immersion in acetone/HA 60/40 wt/wt solution at room temperature, followed by acetone desorption at 50 °C for 60 min, the HA uptake in the film is of 3.3 wt% (Figure 2b). It is worth noting that HA uptake from γ form films provides information on the HA uptake in the amorphous phase. In fact, γ form (differently from δ and ε forms) is a dense form, and sorption of the guest molecules is only allowed in the amorphous phase.

**Figure 3:**
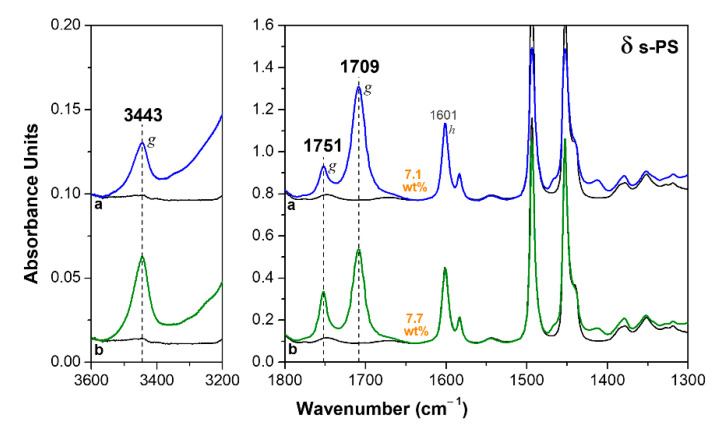
FTIR spectra of an axially oriented δ form s-PS film, after immersion in: (**a**) HA at 70 °C for 12 h; (**b**) 40 wt% solution of HA in acetone at room temperature for 12 h, followed by acetone desorption at 80 °C for 1 h. The uptake of HA in the film (expressed as wt%) is indicated close to the spectra.

FTIR spectra of a 60 µm axially oriented δ form film, after sorption of HA in two different conditions, are shown in Figure 3. The most relevant feature of these spectra is that two additional guest peaks clearly appear: a carbonyl stretching at 1751 cm^−1^, as well as a O−H stretching peak at 3443 cm^−1^, which is in the typical region of carboxylic acids at low concentration [47]. Both peaks are broad and barely detectable for the HA molecules absorbed in the amorphous phase. Based on an analogy with other carboxylic acids [37,38,39,40,41,42], and in agreement with a report from Kaneko and co-workers [34], these new peaks can be attributed to isolated guest molecules, while the peak at 1709 cm^−1^ can be attributed to hydrogen-bonded guest molecules.

In this framework, it is reasonable to assume that isolated and hydrogen-bonded HA molecules (with carbonyl peak at 1751 cm^−1^ and 1709 cm^−1^) are mainly enclosed as guests in the crystalline cavities and in the amorphous phase of the δ form film, respectively.

The HA uptake by the NC δ form film (Figure 3) is much higher than for the γ form film (Figure 2). In fact, by considering the intensity of both carbonyl stretching peaks, after 12 h of immersion in the liquid at 70 °C, the HA uptake is 7.1 wt% (Figure 3a), i.e., ≈4 times higher than for the γ form film, while, after immersion in HA solution in acetone at room temperature, the HA uptake is 7.7 wt% (Figure 3b), i.e., ≈2.5 times higher than for the γ form film subjected to a similar treatment.

**Figure 4:**
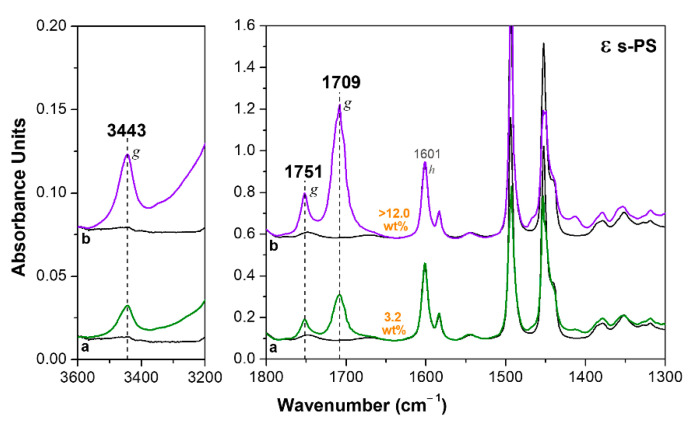
FTIR spectra of an axially oriented ε form s-PS film, after immersion in a 40 wt% solution of HA in acetone at room temperature: (**a**) for 10 min; (**b**) for 2 h. The uptake of HA in the film (expressed as wt%) is indicated close to the spectra.

The HA sorption from NC films exhibiting the ε form is much faster, that is, the NC form in which the empty space is organized as channels [19,23,48,49]. As an example, only 10 min of immersion of a 120 µm thick film in a 40 wt% solution of HA in acetone, at room temperature, are sufficient to reach a HA uptake of 3.2 wt% (Figure 4a). After 2 h of immersion, the HA uptake is higher than 12 wt%.

It is worth adding that the spectra of ε form films (Figure 4), even in the absence of thermal treatments, do not exhibit absorbance peaks of acetone. γ and δ form films, on the contrary, exhibit an intense acetone absorbance carbonyl peak at 1718 cm^−1^ (not shown here) before acetone removal by suitable thermal treatments (at least at 50 °C, as reported above for spectra of Figure 2 and Figure 3). Hence, in the crystalline channels of the ε form, the fast HA sorption does not allow for significant sorption of acetone carrier molecules.

As already observed for the δ form films (Figure 2), for the ε form film, the uptake of the HA generates the additional carbonyl peak at 1751 cm^−1^ and hydroxyl peak at 3443 cm^−1^, which correspond to isolated HA molecules being enclosed in the empty space of the NC ε phase (Figure 4).

Quantitative information on the amount of acid present as a monomer or dimer has been obtained by peak fitting, coupled with DFT calculation of molar extinction coefficients of the carbonyl signals. The peak maxima, observed at 1751 and 1709 cm^−1^, are computed at 1836 and 1772 cm^−1^. While it is expected to have an overestimate of individual experimental frequencies, due to the harmonic approximation used in the calculations, the computed distance of the two carbonyl peaks is also overestimated: 64 cm^−1^ compared with the 42 cm^−1^ experimental entry. As the computed distance agrees more closely with those reported for pure acids [40], the disagreement indicates that the polymer has some effect on the signals of the acid—most likely on the monomer—which, in all s-PS forms, has an experimental value roughly 30 cm^−1^ lower than that reported for octanoic acid [40]; this hints at the formation of a C−H···π bond [50]. For each transition, the calculation gives access to a molar extinction coefficient, and then it is possible to compute the ratio $\frac{\varepsilon_{1709}^{dim}}{\varepsilon_{1751}^{mon}}$ = 2.43, larger than the value of 2 expected for two uncoupled carbonyl groups. A fitting as a sum of Lorentzian peaks was performed on the FTIR spectra recorded with unpolarized light on the samples of Figure 3b and Figure 4a for δ and ε films, respectively (see Supplementary Information for the fitted spectra), as well as on the same s-PS forms without the HA guest. In the region of absorption of the monomer, a feeble peak of the host polymer is always present. Then, spectral subtraction is performed, taking care of the different thicknesses of the films, as estimated by the integrated absorbance of the 1601 cm^−1^ peak. Eventually, the percentages of acid absorbed as monomer are estimated from the ratio of integrated absorbances of the peaks at 1751 and 1709 cm^−1^, corrected for host absorption and the DFT-derived ratio of molar extinction as the following formula:

(3) $$\%\mathrm{mon}=\frac{\frac{1}{2}\frac{\varepsilon_{1709}^{dim}}{\varepsilon_{1751}^{mon}}\left(I_{1751}^{h+\mathrm{HA}}-I_{1751}^{h}\frac{I_{1601}^{h+\mathrm{HA}}}{I_{1601}^{h}}\right)}{I_{1709}^{h+\mathrm{HA}}}$$

where the superscript *h* stands for either δ or ε. Results are given in Table 1.

It is apparent there is a higher amount of HA isolated guest molecules (almost 3 times higher) in the δ phase, i.e., the NC phase presenting isolated cavities, with respect to that absorbed in ε phase, in which the empty space is organized as channels and hence seems to be more suitable to absorb hydrogen-bonded HA molecules than an isolated guest.

HA sorption in the γ form film does not change the diffraction patterns shown in Figure 1A,A’, confirming that (as generally observed for semicrystalline polymers) guest molecules are only absorbed by amorphous phases. Remarkable changes are instead observed, as a consequence of HA sorption, for the WAXD patterns of the NC δ and ε forms, which indicate the formation of CC phases.

**Figure 5:**
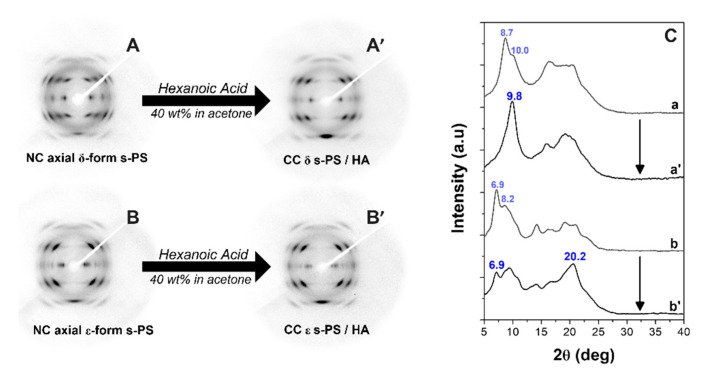
WAXD patterns of axially stretched s-PS films exhibiting the NC δ form before (**A**) and after HA sorption (**A’**) and the NC ε form before (**B**) and after HA sorption (**B’**). The equatorial profiles of these 2D patterns are shown in **(C)** (a, a’, b, b’).

For instance, a comparison between WAXD patterns of the axially oriented δ and ε films of Figure 1, before and after sorption of HA from 40 wt% solution in acetone, with a guest content of nearly 7 and 12 wt%, respectively, is shown in Figure 5.

In particular, for the δ form film, the equatorial peak at 2θ = 8.7° and the shoulder at 2θ ≈ 10° of the triclinic NC δ phase are replaced by a single diffraction peak at 2θ = 9.8°, typical of the disordered CC form [51]. Analogously, for the ε form film, the intensity of the equatorial (110) and (020) peaks at 2θ = 6.9° and 8.2°, respectively, is markedly decreased with respect to the intensity of the first-layer line reflections (mainly that one at 2θ ≈ 20.2°), as is typical of CC ε form films [19,23].

Hence, WAXD patterns of Figure 5 confirm that HA guest molecules are not only absorbed by the amorphous s-PS phase but also included in the crystalline cavities of both δ and ε NC phases, leading to the formation of corresponding CC phases.

### 3.2. Dichroism of FTIR Peaks of HA in Axially Oriented s-PS Films

**Figure 6:**
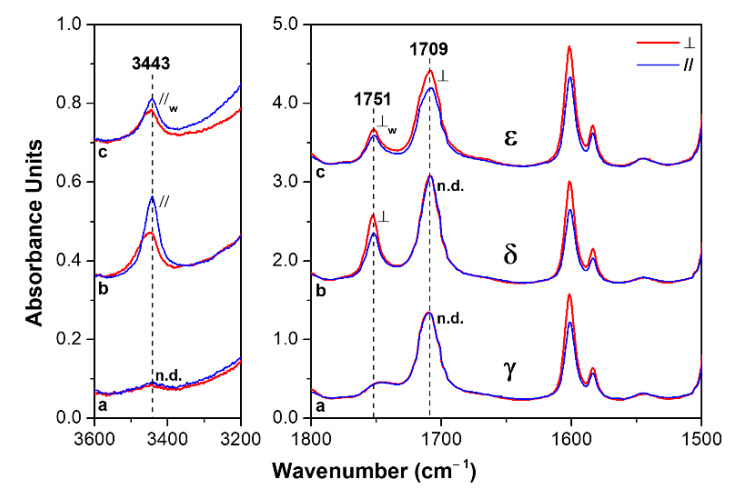
Polarized FTIR spectra as taken with polarization plane parallel (blue lines) and perpendicular (red lines) to the film stretching direction, for two spectral ranges (3600–3200 cm^−1^ and 1800–1500 cm^−1^), for axially oriented s-PS semicrystalline films exhibiting different crystalline phases, after comparable uptake of HA guest molecules: (**a**) γ form; (**b**) δ form: (**c**) ε form.

Polarized FTIR spectra for 3600−3200 cm^−1^ and 1800−1500 cm^−1^ regions of axially oriented γ, δ and ε s-PS films, which exhibit similar intensity of the guest peak at 1709 cm^−1^, are shown in Figure 6. The HA uptake is close to 3.5 wt% for the γ form film while it is close to 7 wt% for both δ and ε form films.

The intensity differences between peaks collected with polarization planes parallel or perpendicular to the film-stretching direction (blue and red spectra, respectively) clearly show the presence of dichroism of the peaks of the host polymer. The orientation factor *S_h_*, as evaluated on the basis of the dichroism of the crystalline peak at 572 cm^−1^, is for the three films in the range 0.83−0.88.

As for the HA guest peaks, dependent on the polymer crystalline form, some dichroism can be observed for the carbonyl stretching peaks at 1751 and 1709 cm^−1^, as well as for the hydroxyl peak at 3443 cm^−1^. In particular, the dichroism is absent for molecules absorbed by the γ form film (Figure 6a), thus confirming that HA molecules are not absorbed by the dense γ phase, but only absorbed in the poorly oriented amorphous phase.

As for the δ form film (Figure 6b), significant dichroism is observed for the vibrational peaks corresponding to isolated HA molecules (*S_g_*_,1751_ = −0.11 and *S_g_*_,3443_ = +0.24), while no dichroism is observed for the hydrogen-bonded HA molecules. This confirms the previous hypothesis that isolated HA molecules are guests of the more oriented crystalline phase, while hydrogen-bonded HA molecules are guests of the less oriented amorphous phase [52,53].

The dichroic behavior of peaks of HA guest molecules absorbed in the NC ε form films is completely different (Figure 6c). In fact, the dichroism of the carbonyl peak of hydrogen-bonded HA molecules (*S_g_*_,1709_ = −0.06) is similar to those of the peaks corresponding to isolated guest molecules (*S_g_*_,1751_ = −0.07 and *S_g_*_,3443_ = +0.10). These data can be rationalized by assuming that the structural channels of the ε form are able to host not only isolated HA molecules but also HA dimers.

The sorption of HA, both as a monomer and dimer, in the crystalline channels of the ε form can also rationalize the exceptionally high and fast HA uptake from these semicrystalline films (see previous section).

**Figure 7:**
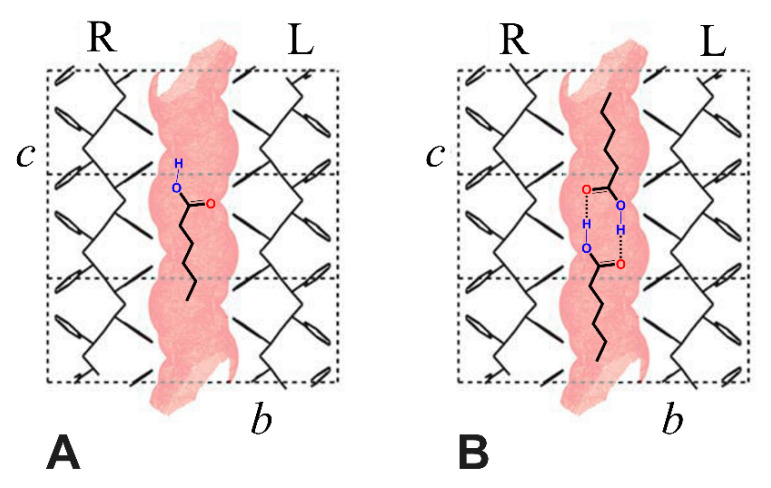
Schematic presentation of isolated (**A**) and dimeric (**B**) HA species in the crystalline channels of the ε form. The parallelism of the aliphatic chain with respect to the chain axis and the minimum energy conformation of the carboxylic groups (C=O eclipsed) are able to rationalize the orientations of O−H and C=O groups, preferentially parallel and perpendicular to the polymer chain crystalline axes, respectively, as established by FTIR linear dichroism measurements.

It is worth noting that the dichroism of the O−H stretching for HA isolated guest in both δ and ε phases is positive, indicating a preferential orientation of the O−H bond parallel to the crystalline polymer chain axis, independent of the kind of NC phase. Moreover, the dichroism of the C=O stretching for the isolated HA guest in the δ form, and for both isolated and dimeric HA guests in the ε form, are always negative, indicating a preferential orientation of the C=O bond perpendicular to the crystalline polymer chain axis. These orientations of the O–H and C=O groups, preferentially parallel and perpendicular to the polymer chain axis, respectively, can be easily rationalized for HA guest molecules in the crystalline channels of the ε form. In fact, these preferential orientations can be anticipated on the basis of two obvious structural features: (i) parallelism of the aliphatic chains (in their trans-planar conformation) with respect to the chain axes of the polymer crystalline phase; (ii) minimum energy conformation of aliphatic carboxylic acids, which exhibit the double bonded carboxyl-oxygen atom in an eclipsed position with respect to the β carbon atom [54]. This is shown for monomeric and dimeric HA species in the crystalline channels of the ε form by the schematic drawings of Figure 7A,B, respectively.

**Figure 8:**
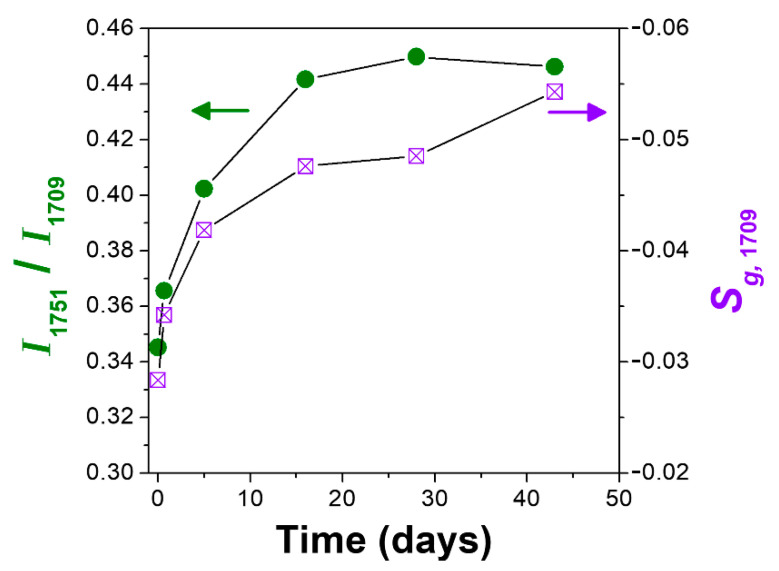
Intensity ratio of the two carbonyl peaks *I*_1751_/*I*_1709_, corresponding to isolated and hydrogen bonded HA guest molecules (left scale and green circles), and dichroism of the 1709 cm^−1^ peak (right scale and violet squares) versus the guest desorption time, for the ε form film.

Additional relevant information comes by studies of HA desorption from s-PS CC films, e.g., at room temperature in air. In fact, the ratio between the two peaks corresponding to isolated and hydrogen-bonded HA molecules (*I*_1751_/*I*_1709_) increases with desorption time. This phenomenon, which occurs for both δ and ε form films, is shown for the ε form film (left scale in Figure 8) and can easily be rationalized by guest desorption from amorphous s-PS phases occurring faster than from CC phases [1,31,32].

The increase in the dichroic ratio of the 1709 cm^−1^ peak (right scale in Figure 8), with guest desorption time, for the ε form film is also interesting. This phenomenon can, again, be rationalized by the easier desorption of hydrogen-bonded HA from the less-oriented amorphous phase with respect to hydrogen-bonded HA dimer from the more-oriented ε phase.

## 4. Conclusions

The sorption of a carboxylic acid (hexanoic acid, HA) is definitely higher and faster in s-PS films with NC δ and ε phases than for s-PS films with the dense γ phase. HA sorption is particularly fast for ε form films exhibiting crystalline empty channels.

The sorption of HA in s-PS films with NC δ and ε phases leads to guest uptake not only in their amorphous phases, but also in the cavities and channels of their crystalline phases, respectively. In fact, the formation of δ and ε s-PS/HA CC phases is shown by changes in reflection intensities in WAXD fiber patterns.

The appearance of two dichroic FTIR peaks (a carbonyl and a hydroxyl stretching at 1751 cm^−1^ and 3443 cm^−1^, respectively) indicate that both δ and ε NC phases are able to include HA as isolated guest molecules. The dichroism of the carbonyl peak corresponding to hydrogen-bonded molecules (at 1709 cm^−1^), observed only for ε form films, indicates that HA dimers are also included in the crystalline channels of the ε phase. Moreover, the sign of dichroism of the O−H stretching and C=O stretching peaks indicates that the orientations of O−H and C=O bonds are preferentially parallel and perpendicular to the crystalline polymer chain axes, respectively. These functional group orientations can easily be rationalized for both monomeric and dimeric HA guest molecules in the crystalline channels of the ε form.

The inclusion of HA molecules in the channels of the ε phase, not only as isolated molecules, but also as dimers, is possibly the molecular origin of the much faster HA uptake from ε form s-PS films.

This is the first reported case of a polymeric CC phase including a hydrogen-bonded dimeric species. As this phenomenon occurs in the NC ε form, exhibiting continuous crystalline channels parallel to the polymer chain axis, our results suggest the possibility of inclusion in these channels of continuous molecular chains formed by hydrogen bonded dicarboxylic acids. This can open new perspectives in the use of CC polymer films as functional materials.

## Figures and Tables

**Table 1:** Integrated absorbances (in cm^−1^) obtained by non-linear least square fitting of the spectra as sum of Lorentzian peaks, and the derived percentage of isolated guest HA molecules.

Form	*I*_1601_	*I*_1709_	*I*_1751_	%mon
δ	17.8	-	2.0	
δ + HA	15.2	30.7	12.7	43
ε	29.2	-	6.3	
ε + HA	33.2	35.6	11.2	14

## Data Availability

Not applicable.

## Supplementary Materials

The following are available online at https://www.mdpi.com/article/10.3390/polym13193330/s1. Figure S1: Fit of a portion of the FTIR spectrum of form of s-PS as a sum of Lorentzian peaks. The magenta line of the model is best appreciated by zooming the figure. Individual Lorentzian peaks are displayed as dashed red lines. The residuals are shown as a black line below the spectrum, flanked by two dashed red lines displaced above or below by the a posteriori standard deviation, computed as the square root of the sum of squares of the residuals divided by the number of degrees of freedom, Figure S2: Fit of a portion of the FTIR spectrum of form of s-PS as a sum of Lorentzian peaks. The magenta line of the model is best appreciated by zooming the figure. Individual Lorentzian peaks are displayed as dashed red lines. The residuals are shown as a black line below the spectrum, flanked by two dashed red lines displaced above or below by the a posteriori standard deviation, computed as the square root of the sum of squares of the residuals divided by the number of degrees of freedom.
