# Supplementary material for: Monomeric and Dimeric Carboxylic Acid in Crystalline Cavities and Channels of Delta and Epsilon Forms of Syndiotactic Polystyrene
Source: Polymers (Basel). 2021 Sep 29;13(19):3330. doi: 10.3390/polym13193330 (PMC8512363; doi:10.3390/polym13193330)
Supplement: Supplementary file 1 [file polymers-13-03330-s001.zip › polymers-1399300-supplementary.pdf]

Monomeric and dimeric carboxylic acid in crystalline cavities and channels of δ and ε forms of syndiotactic polystyrene

Supplementary Information

Antonietta Cozzolino, Guglielmo Monaco, Christophe Daniel, Paola Rizzo*, Gaetano Guerra*

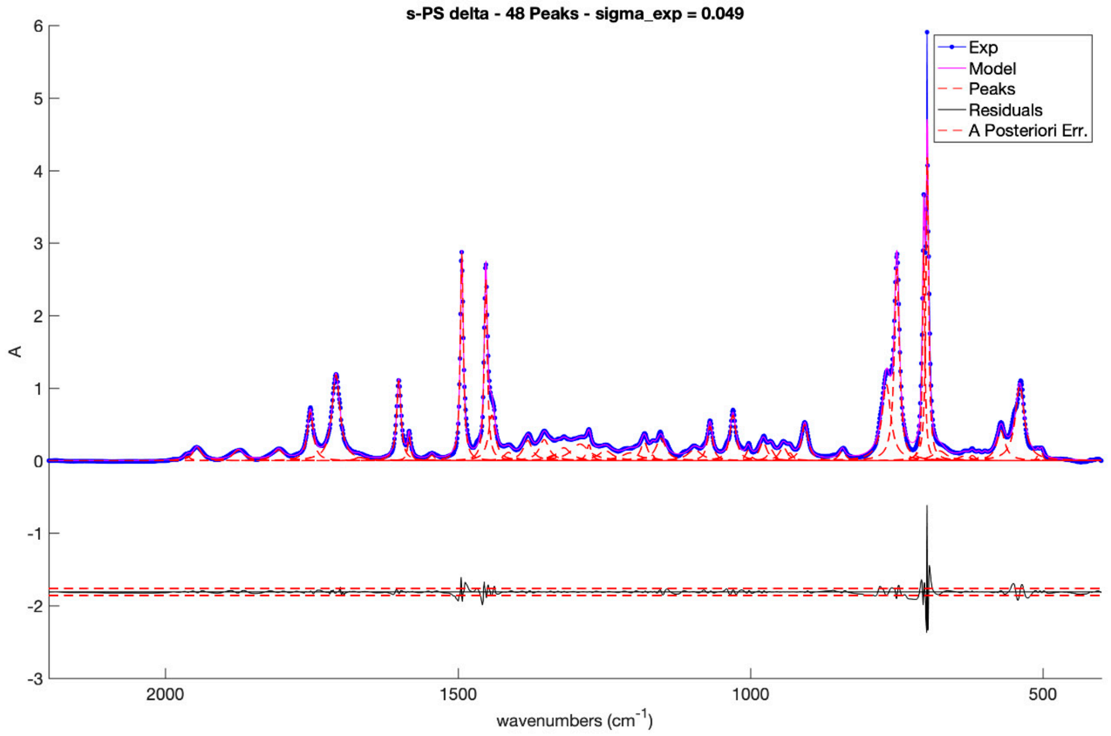

Figure S1. Fit of a portion of the FTIR spectrum of δ form of s-PS as a sum of Lorentzian peaks. The magenta line of the model is best appreciated by zooming the figure. Individual Lorentzian peaks are displayed as dashed red lines. The residuals are shown as a black line below the spectrum, flanked

by two dashed red lines displaced above or below by the a posteriori standard deviation, computed as the square root of the sum of squares of the residuals divided by the number of degrees of freedom.

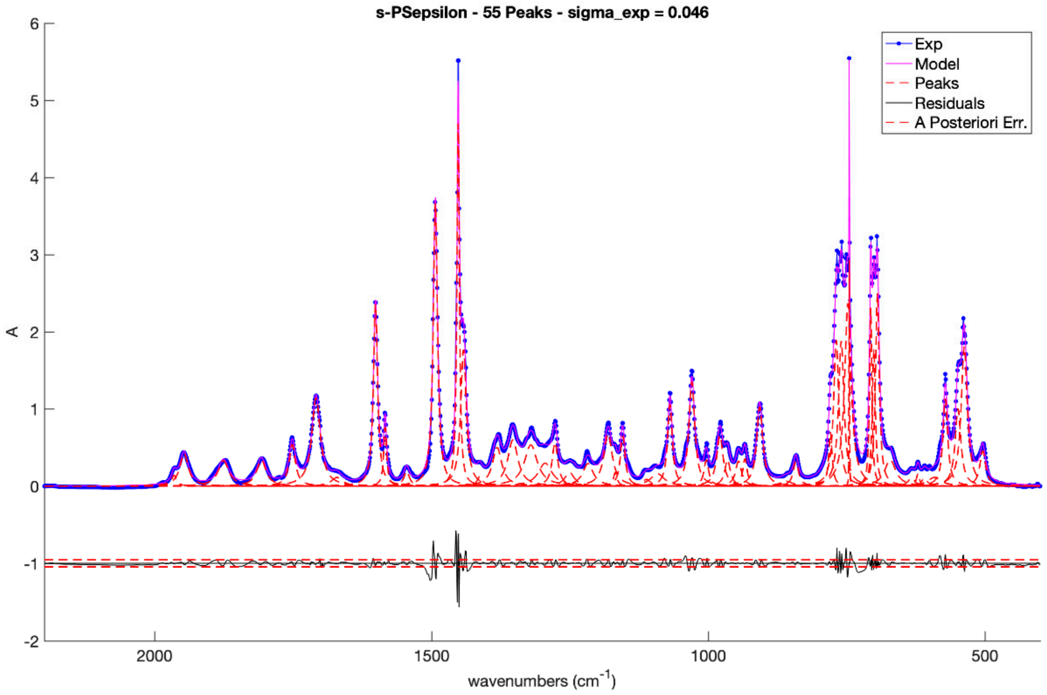

Figure S2. Fit of a portion of the FTIR spectrum of ε form of s-PS as a sum of Lorentzian peaks. The magenta line of the model is best appreciated by zooming the figure. Individual Lorentzian peaks are displayed as dashed red lines. The residuals are shown as a black line below the spectrum, flanked by two dashed red lines displaced above or below by the a posteriori standard deviation, computed as the square root of the sum of squares of the residuals divided by the number of degrees of freedom.
